# Clinical characteristics of pyogenic liver abscess with and without biliary surgery history: a retrospective single-center experience

**DOI:** 10.1186/s12879-024-09378-x

**Published:** 2024-05-10

**Authors:** Yuxin Lin, Yifan Chen, Weiyin Lu, Yu Zhang, Rongqian Wu, Zhaoqing Du

**Affiliations:** 1 Department of Hepatobiliary Surgery, Shaanxi Provincial People’s Hospital, 256 West Youyi Road, Xi’an, Shaanxi Province 710068 China; 2 Shanghai Institute of Hematology, State Key Laboratory of Medical Genomics, National Research Center for Translational Medicine at Shanghai, Ruijin Hospital Affiliated to Shanghai Jiao Tong University School of Medicine, 197 Rui Jin Er Road, Shanghai, 200025 China; 3 Department of Gastroenterology, Peking University Third Hospital, Beijing, 100191 China; 4 Xi’an Medical University, Xi’an, Shaanxi 710021 China; 5 National-Local Joint Engineering Research Center for Precision Surgery & Regenerative Medicine, Institute of Advanced Surgical Technology and Engineering, First Affiliated Hospital of Xi’an Jiaotong University, Xi’an, Shaanxi Province 710061 China; 6 National Engineering Research Center for Miniaturized Detection Systems, College of Life Science, Northwest University of Xi’an, Xi’an, Shaanxi Province 710069 China

**Keywords:** Biliary surgery, Pyogenic liver abscess, Bilioenteric anastomoses

## Abstract

**Background & aims:**

Pyogenic liver abscess (PLA) is a common hepatobiliary infection that has been shown to have an increasing incidence, with biliary surgery being identified as a trigger. Our aim was to investigate the clinical characteristics and treatments of PLA patients with and without a history of biliary surgery (BS).

**Methods:**

The study included a total of 353 patients with PLA who received treatment at our hospital between January 2014 and February 2023. These patients were categorized into two groups: the BS group (*n* = 91) and the non-BS group (*n* = 262). In the BS group, according to the anastomosis method, they were further divided into bilioenteric anastomoses group (BEA, *n* = 22) and non-bilioenteric anastomoses group (non-BEA, *n* = 69). Clinical characteristics were recorded and analyzed.

**Results:**

The percentage of PLA patients with BS history was 25.78%. The BS group exhibited elevated levels of TBIL and activated APTT abnormalities (*P* = 0.009 and *P* = 0.041, respectively). Within the BS group, the BEA subgroup had a higher prevalence of diabetes mellitus (*P* < 0.001) and solitary abscesses (*P* = 0.008) compared to the non-BEA subgroup. *Escherichia coli* was more frequently detected in the BS group, as evidenced by positive *pus* cultures (*P* = 0.021). The BS group exhibited reduced treatment efficacy compared to those non-BS history (*P* = 0.020). Intriguingly, the BS group received a higher proportion of conservative treatment (45.05% vs. 21.76%), along with reduced utilization of surgical drainage (6.59% vs. 16.41%).

**Conclusions:**

Patients with BS history, especially those who have undergone BEA, have an increased susceptibility to PLA formation without affecting prognosis.

## Lay summary

The present study aimed to explore the disparities in clinical characteristics and treatment approaches between patients with pyogenic liver abscess (PLA) who had undergone biliary surgery and those without such surgical history.

## Introductions

Pyogenic liver abscess (PLA) is a prevalent yet potentially life-threatening hepatobiliary infection that has demonstrated an increasing incidence. Approximately 40% of cases can be attributed to biliary diseases following infection [1]. Biliary surgery can contribute to the rise in PLA occurrence due to alterations in biliary anatomy and potential complications, such as post-biliary surgery cholangitis and hepatic artery thrombosis [2–4]. Bilioenteric anastomoses (BEA) are frequently performed surgical procedures aimed at preventing biliary strictures, which result in the absence of the sphincter of *Oddi*. This can potentially lead to the reflux of bowel bacteria into the bile ducts and even liver, ultimately causing the formation of liver abscesses [5]. Biliary obstruction and strictures following biliary surgery also play crucial roles in the development of PLA [6]. It has been established that patients with altered biliary anatomy tend to experience ipsilateral biliary infections [7]. Additionally, hepatic artery thrombosis secondary to biliary procedures can contribute to PLA through liver ischemia resulting from obstructed bile ducts after surgery [6].

Besides being one of the triggering factors for PLA, a history of biliary surgery has been proven to significantly impact both the clinical presentation and prognosis of patients, including differences in pathogenic bacteria and mortality rates [8]. Additionally, PLA patients with a history of hepatojejunostomy tend to have lower body mass index (BMI) and are more prone to renal failure [6]. The specific type of biliary surgery, particularly BEA, also plays a role in the development and characteristics of PLA. For instance, hepatojejunostomy carries a higher risk of infection leading to PLA compared to duct-to-duct biliary anastomosis [9]. The development of bacterial colonization in the fragile liver parenchyma of patients with a history of biliary surgery is promoted due to factors such as immunosuppression (e.g., diabetes or chemotherapy) and general health impairment [4]. However, there has been limited research investigating the clinical characteristics between patients with PLA who have a history of biliary surgery compared to those without. Therefore, we conducted a single-center study aimed at improving the diagnosis and prognosis of PLA patients undergoing biliary surgery and bilioenteric anastomosis.

## Patients and methods

### Patients

This retrospective single-center study included 422 patients primarily diagnosed with pyogenic liver abscess (PLA) in our hospital from January 2014 to February 2023. All PLA cases were defined based on clinical features, imaging and laboratory results, as well as blood and *pus* cultures of the lesion material. A total of 69 patients were excluded based on the following criteria: those diagnosed with parasitic, fungal, amoebic abscesses or tumors; those who developed PLA as a complication; those whose primary cause of hospitalization was not PLA; those who did not complete their hospitalization at our hospital; and those with incomplete medical records or lacking a 90-day follow-up. Finally, a total of 353 patients were included in our study. Initially, the patients were categorized into two groups based on their history of biliary surgery (BS) or non-biliary surgery (non-BS). We collected information regarding the specific types of biliary surgeries performed in the BS group (Table 1). Among patients with BS history, they were further divided into the BEA group (22 cases) and the non-BEA group (69 cases). The 22 BEA patients included 1 case of pancreatic duct stones, 6 cases of intrahepatic bile duct stones, 1 case of bile duct stenosis, 2 cases of common bile duct injury, 2 cases of congenital choledochal cyst, and 10 cases of recurrent common bile duct stones. Additionally, we conducted a comparative analysis between conservative and non-conservative treatments for PLA patients with a history of BS. It is important to note that this study strictly adhered to the principles outlined in the Treaty of Helsinki. The Medical Ethics Committee of Shaanxi Provincial People’s Hospital agreed to waive the written informed consent due to the retrospective nature of this study.

**Table 1:** Types of biliary surgery history in this study

Details of biliary surgery history	Cases (*n* = 91)
Bile intestinal anastomosis	22 (24.18%)
Intrahepatic bile duct stone	10 (10.99%)
Simple cholecystectomy	37 (40.66%)
Cholecystectomy + CBD	13 (14.29%)
ERCP + EST	7 (7.69%)
Liver transplantation	6 (6.59%)
Choledocholithotomy	5 (5.49%)
Biliary metallic stent implantation	1 (1.10%)

### Data collection

The data, including patient details, was collected from the hospital’s electronic medical records system. The general data we retrieved included age, gender, underlying conditions, and symptoms. Laboratory results obtained at admission consisted of blood routine, liver function tests, coagulation profiles, renal function tests, as well as blood and *pus* culture data (including those who had either or both). Additionally, information regarding treatments administered, antibiotic usage patterns and outcomes were also acquired. We utilized ultrasonography and computerized tomography (CT) to quantify the diameter, number, gas-forming ability, and location of abscesses. In our study, curation was defined as the presence of light spots similar to those in normal livers under ultrasound, accompanied by complete disappearance of the dark liquid area in the liver, normalization of laboratory abnormalities, and resolution of clinical symptoms and signs, particularly fever and hepatomegaly. Improvement referred to either unchanged or reduced abnormal imaging features as well as alleviation of clinical symptoms and signs.

### Follow-up

The average duration of follow-up, which lasted for at least 3 months post-discharge, was 102 (50–180) days. Comprehensive follow-up data were collected from all patients in July 2023, with a primary focus on disease status and the need for retreatment.

### Statistical analysis

The statistical analyses were conducted using SPSS 23.0 software (IBM Corporation, Armonk, NY, USA). Frequency and percentage were used to express categorical variables, while median (min-max) was employed to describe continuous variables. Additionally, the difference between the two groups was calculated by applying Student’s t-test or Wilcoxon test for continuous variables and chi-squared test or Fisher’s exact test for categorical data. A significance level of 0.05 was utilized.

## Results

### Patient demographic and clinical characteristics

There were 353 patients diagnosed with PLA in our study. As indicated in Table 2, a total of 149 (42.21%) patients were aged ≥ 60 years, and among the entire patient cohort, there were 212 (60.06%) males and 141 (39.94%) females. Furthermore, a history of smoking was present in 98 (27.76%) individuals, while alcohol consumption history was reported by 63 (17.85%) patients. Hypertension and diabetes mellitus had developed in 71 (20.11%) and 102 (28.90%) patients respectively, whereas cardiovascular diseases had manifested in 19 (5.38%). Cirrhosis was observed in only 11 (3.12%) cases, and pleural effusion was documented in the medical records of 32 enrolled patients (9.07%). Pneumonia affected a total of 18 individuals (5.10%), while pericardial effusion at admission occurred rarely with only two cases (0.57%). The predominant symptom observed among patients was fever (87.82%), followed by chills (57.51%), abdominal pain (44.76%), and nausea or vomiting (22.66%). Additionally, jaundice was present in 16 (4.53%) patients, while fatigue was reported by 60 (17.00%) individuals. On admission day, 178 (50.42%) patients exhibited an abnormal increase in leukocyte count. The average diameter of the abscess was measured at 6.10 cm (range: 0.60–17.30), and the majority of patients (77.62%) developed a single abscess. The right liver lobe is the most prevalent site of abscess formation in this cohort, accounting for 62.61% of cases, followed by the left liver lobe at 15.30%. Additionally, bilateral abscesses were observed in 38 (10.76%) cases. Gas formation was also noted in 43 (12.18%) patients.

**Table 2:** Clinical characteristics of 353 PLA patients with biliary surgery (BS) and non-biliary surgery history presented at admission

Values	Median (range)/*n* (percentage)	PLA with BS history	PLA with non-BS history	*P* value
N	353	91	262	
Age (≥ 60)	149 (42.21%)	46 (50.55%)	103 (39.31%)	0.062
Gender (male/female)	212 (60.06%)/141 (39.94%)	43 (47.25%)/48 (52.75%)	169 (64.50%)/93 (35.50%)	**0.004**
Underlying conditions				
Smoking	98 (27.76%)	20 (21.98%)	78 (29.77%)	0.153
Drinking	63 (17.85%)	12 (13.19%)	51 (19.47%)	0.178
Hypertension	71 (20.11%)	16 (17.58%)	55 (20.99%)	0.484
Diabetes Mellitus	102 (28.90%)	46 (50.55%)	56 (21.37%)	**< 0.001**
Cardiovascular diseases	19 (5.38%)	5 (5.49%)	14 (5.34%)	0.830
Cirrhosis	11 (3.12%)	6 (6.59%)	5 (1.91%)	0.062
Pleural effusion	32 (9.07%)	24 (26.37%)	8 (3.05%)	**< 0.001**
Pneumonia	18 (5.10%)	3 (3.30%)	15 (5.73%)	0.528
Pericardial effusion	2 (0.57%)	0 (0.00%)	2 (0.76%)	1.000
Symptoms				
Fever	310 (87.82%)	84 (92.31%)	226 (86.26%)	0.129
Chills	203 (57.51%)	52 (57.14%)	151 (57.63%)	0.935
Abdominal pain	158 (44.76%)	44 (48.35%)	114 (43.51%)	0.424
Jaundice	16 (4.53%)	4 (4.40%)	12 (4.58%)	0.826
Nausea or vomiting	80 (22.66%)	21 (23.08%)	59 (22.52%)	0.913
Fatigue	60 (17.00%)	15 (16.48%)	45 (17.18%)	0.880
Laboratory tests at admission				
WBC > 10 × 10^9^/L	178 (50.42%)	41 (45.05%)	137 (52.29%)	0.234
HGB < 120 g/L	202 (57.22%)	56 (61.54%)	146 (55.73%)	0.334
PLT < 100 × 10^9^/L	43 (12.18%)	11 (12.09%)	32 (12.21%)	0.975
ALT > 40 U/L	165 (46.74%)	39 (42.86%)	126 (48.09%)	0.389
AST > 40 U/L	106 (30.03%)	28 (30.77%)	78 (29.77%)	0.858
ALP > 120 μmol/L	236 (66.86%)	62 (68.13%)	174 (66.41%)	0.764
GGT > 60 U/L	277 (78.47%)	71 (78.02%)	206 (78.63%)	0.904
TBIL > 17 μmol/L	134 (37.96%)	45 (49.45%)	89 (33.97%)	**0.009**
ALB < 35 g/L	265 (75.07%)	67 (73.63%)	198 (75.57%)	0.712
PT > 17 s	25 (7.08%)	9 (9.89%)	16 (6.11%)	0.225
APTT > 45 s	38 (10.76%)	15 (16.48%)	23 (8.78%)	**0.041**
BUN > 7.2 mmol/L	53 (15.01%)	15 (16.48%)	38 (14.50%)	0.649
Cr > 97 μmol/L	24 (6.80%)	6 (6.59%)	18 (6.87%)	0.928
Abscess diameter (cm)	6.10 (0.60–17.30)	5.80 (0.60–16.10)	6.30 (1.04–17.30)	0.146
Abscess number				
Solitary abscess	274 (77.62%)	67 (73.63%)	207 (79.01%)	0.289
Multiple abscess	79 (22.38%)	24 (26.37%)	55 (20.99%)	
Gas forming	43 (12.18%)	16 (17.58%)	27 (10.31%)	**0.067**
Abscess site				
Both left and right	38 (10.76%)	11 (12.09%)	27 (10.31%)	0.452
Left lobe	54 (15.30%)	10 (10.99%)	44 (16.79%)	
Right lobe	221 (62.61%)	57 (62.64%)	164 (62.60%)	
Other sites	40 (11.33%)	13 (14.29%)	27 (10.31%)	

The results presented in Table 3 demonstrate that a total of 222 *pus* cultures were collected, with 70 patients (31.53%) yielding negative findings. Among the positive results, *Klebsiella pneumoniae* and *Escherichia coli* were identified as the two most prevalent pathogens in this study, accounting for 43.24% and 7.21%, respectively. Furthermore, a total of 166 blood culture samples were obtained, with negative results observed in 127 cases (76.51%). Notably, *Klebsiella pneumoniae* was also found to be the predominant pathogen among the positive blood cultures (13.25%), followed by *Streptococcus* (3.01%). In Table 4, it was documented that percutaneous drainage was performed on 206 (58.36%) patients, while surgical drainage was received by 49 (13.88%), and conservative treatment was given to 98 (27.76%) patients. Antibiotic therapy consisted of the third generation cephalosporins in 230 cases (65.16%), followed by carbapenems in 143 cases (40.51%). The majority of patients showed clinical improvement or were cured during hospitalization (73.09% and 20.68%, respectively), with a median time for temperature normalization of 6 days (range:0–40). Finally, the median expenses for patients ranged from $57.69 to $29,275.83 and retreatment within 3 months occurred in only four cases (1.13%).

**Table 3:** Blood and *pus* culture results in PLA patients with BS and non-BS history

	Total	PLA with BS history	PLA with non-BS history	*P* value
*Pus* culture (n, %)	222	52	170	
*Klebsiella pneumonia*	96 (43.24%)	21 (40.38%)	75 (44.12%)	0.634
*Streptococcus spp.*	8 (3.60%)	1 (1.92%)	7 (4.12%)	0.751
*Escherichia coli*	16 (7.21%)	8 (15.38%)	8 (4.71%)	**0.021**
*Enterococcus spp.*	7 (3.15%)	2 (3.85%)	5 (2.94%)	0.899
*Candida spp.*	1 (0.45%)	0 (0.00%)	1 (0.59%)	1.000
*Staphylococcus*	2 (0.90%)	0 (0.00%)	2 (1.18%)	1.000
*Pseudomonas aeruginosa*	2 (0.90%)	1 (1.92%)	1 (0.59%)	0.414
*Proteus spp.*	1 (0.45%)	1 (1.92%)	0 (0.00%)	0.234
Others	5 (2.25%)	1 (1.92%)	4 (2.35%)	0.725
Multiple bacteria	14 (6.31%)	8 (15.38%)	6 (3.53%)	**0.006**
Negative results	70 (31.53%)	9 (17.31%)	61 (35.88%)	**0.012**
Blood culture (n, %)	166	41	125	
*Klebsiella pneumonia*	22 (13.25%)	1 (2.44%)	21 (16.80%)	**0.019**
*Escherichia coli*	3 (1.81%)	0 (0.00%)	3 (2.40%)	1.000
*Streptococcus*	5 (3.01%)	1 (2.44%)	4 (3.20%)	0.780
*Staphylococcus*	2 (1.20%)	0 (0.00%)	2 (1.60%)	1.000
Others	3 (1.81%)	2 (4.88%)	1 (0.80%)	0.151
Multiple bacteria	4 (2.41%)	3 (7.32%)	1 (0.80%)	**0.047**
Negative results	127 (76.51%)	34 (82.93%)	93 (74.40%)	0.264

**Table 4:** Treatment and outcomes of the PLA patients with BS and non-BS history

Variables	Median (range)/*n* (percentage)	PLA with BS history	PLA with non-BS history	*P* value
Treatment				
Percutaneous drainage	206 (58.36%)	44 (48.35%)	162 (61.83%)	**< 0.001**
Surgical drainage	49 (13.88%)	6 (6.59%)	43 (16.41%)	
Conservative treatment	98 (27.76%)	41 (45.05%)	57 (21.76%)	
Antibiotic use				
The 3^rd^ generation of cephalosporin	230 (65.16%)	51 (56.04%)	179 (68.32%)	**0.034**
Carbapenems	143 (40.51%)	43 (47.25%)	100 (38.17%)	0.128
Nitroimidazoles	89 (25.21%)	24 (26.37%)	65 (24.81%)	0.767
Fluoroquinolone	38 (10.76%)	4 (4.40%)	34 (12.98%)	**0.023**
Glycopeptide	12 (3.40%)	5 (5.49%)	7 (2.67%)	0.345
Outcomes				
Cured	258 (73.09%)	65 (71.43%)	193 (73.66%)	**0.020**
Improved	73 (20.68%)	15 (16.48%)	58 (22.14%)	
Useless	22 (6.23%)	11 (12.09%)	11 (4.20%)	
Time for temperature normalization (days)	6 (0–40)	5 (0–28)	6 (0–40)	0.165
Hospital stay (days)	13 (1–52)	12 (2–52)	13 (1–51)	0.611
Total Hospitalization expenses (dollars)	3384.71 (57.69–29,275.83)	3230.77 (341.72–29,275.83)	3420.80 (57.69–19,468.29)	0.644
Retreatments in 3 months	4 (1.13%)	2 (2.20%)	2 (0.76%)	0.590

### Associations between biliary surgery history and patients’ characteristics

In this study, a total of 353 patients were initially categorized into two groups based on their history of biliary surgery (BS) and non-biliary surgery (non-BS). The non-BS group comprised 262 patients, while the BS group consisted of 91 patients. Among the BS group, simple cholecystectomy was performed in 37 cases (40.66%), followed by bile intestinal anastomosis in 24.18% and cholecystectomy with common bile duct exploration in 14.29% (Table 1). As indicated in Table 2, there were no significant differences observed in terms of age, most underlying conditions, and all recorded symptoms between the BS group and the non-BS group. However, it was noted that a higher proportion of male patients (*P* = 0.004) with a history of biliary surgery presented along with comorbidities such as diabetes mellitus (*P* < 0.001) and pleural effusion (*P* < 0.001). Furthermore, it is evident that the BS group had a higher prevalence of abnormalities in total bilirubin levels (*P* = 0.009) and APTT values (*P* = 0.041). Additionally, an interesting finding emerged indicating similar occurrences of gas-forming conditions regarding abscess size, number, and location between both groups.

Table 3 demonstrates that patients without a history of biliary surgery had a lower incidence of positive *pus* culture results (*P* = 0.012). Additionally, cases with a history of biliary surgery were more likely to have multiple bacterial infections in both *pus* culture (*P* = 0.006) and blood culture (*P* = 0.047). Furthermore, the outcome of *pus* culture indicated that patients in the BS group had a higher prevalence of *Escherichia coli* infection (*P* = 0.021), while the outcome of blood culture showed that patients in the non-BS group had a higher prevalence of *Klebsiella pneumonia* infection (*P* = 0.019).

In Table 4, the choice of treatments between patients with and without a BS history varied a lot (*P* < 0.001). Although percutaneous drainage was the preferred treatment option for both groups, patients with a BS history received more conservative management (45.05% vs. 21.76%) and underwent fewer surgical drainages (6.59% vs. 16.41%). Regarding antibiotic usage, patients with a BS history were less likely to be prescribed third-generation cephalosporins and fluoroquinolones compared to those without a BS history (*P* = 0.034 and *P* = 0.023, respectively). Additionally, there were significant differences in outcomes between the two groups (*P* = 0.020). The non-BS group demonstrated an improvement in condition for 22.14% of cases, whereas only 16.48% of cases in the BS group showed improvement. Conversely, treatments reported as ineffective accounted for 12.09% in the latter group compared to just 4.20% in the former group. However, no significant disparities were observed regarding time required for temperature normalization, duration of hospital stay, total expenses incurred, and retreatment within 3 months between the two groups. It is important to note that improvement refers to the consistent reduction or disappearance of hyperechoic spots, as seen in normal livers under ultrasound, and the resolution of hypoechoic areas within the liver, along with alleviation of clinical symptoms and signs; however, some abnormalities may still persist. Uselessness implies no change in abnormal imaging features and clinical symptoms and signs, particularly fever and hepatomegaly observed in our study.

### Subgroups analysis in PLA patients with BS history

Patients with a history of biliary surgery were categorized into two subgroups based on whether they had undergone bilioenteric anastomoses (BEA). 22 patients were included in BEA group and 69 patients were included in non-BEA group. The non-BEA group consisted of patients who had received other types of biliary surgery instead of bilioenteric anastomoses (Table 5). Although both groups exhibited similarities in terms of age, gender, underlying conditions, symptoms, and laboratory tests, the BEA group demonstrated a higher incidence of diabetes mellitus (*P* < 0.001). There is no significant variation observed between the two groups in terms of abscess size, site, and gas-forming condition. However, there was a substantial difference in the number of abscesses between the groups. The BEA group exhibited predominantly solitary lesions, surpassing the other group (95.45% vs 66.67%, *P* = 0.008). No significant differences were found between the two groups in terms of treatment choice, although the non-BEA group exhibited a higher likelihood of opting for carbapenems compared to the BEA group (*P* = 0.041). Both groups also demonstrated similar clinical outcomes, time required for temperature normalization, duration of hospital stay, total expenses incurred during hospitalization, and rates of retreatment within 3 months (*P* = 0.056).

**Table 5:** The clinical characteristics of PLA patients with BS history between bilioenteric anastomoses (BEA) and non-bilioenteric anastomoses (non-BEA) group

Variables	PLA with BEA history (*n* = 22)	PLA with non-BEA history (*n* = 69)	*P* value
Age (≥ 60)	11 (50.00%)	34 (49.28%)	0.953
Gender (male/female)	10 (45.45%)/12 (54.55%)	33 (47.83%)/36 (52.17%)	0.846
Underlying condition			
Smoking	3 (13.64%)	17 (24.64%)	0.430
Drinking	2 (9.09%)	10 (14.49%)	0.772
Hypertension	3 (13.64%)	14 (20.29%)	0.702
Diabetes Mellitus	22 (100.00%)	23 (33.33%)	**< 0.001**
Cardiovascular diseases	0 (0.00%)	5 (7.25%)	0.446
Cirrhosis	0 (0.00%)	6 (8.70%)	0.348
Pleural effusion	6 (27.27%)	18 (26.09%)	0.912
Pneumonia	0 (0.00%)	2 (2.90%)	1.000
Symptoms			
Fever	22 (100.00%)	62 (89.86%)	0.273
Chills	12 (54.55%)	40 (57.97%)	0.777
Abdominal pain	12 (54.55%)	32 (46.38%)	0.504
Jaundice	2 (9.09%)	2 (2.90%)	0.245
Nausea or vomiting	8 (36.36%)	13 (18.84%)	0.089
Fatigue	1 (4.55%)	14 (20.29%)	0.161
Laboratory tests at admission			
WBC > 10 × 10^9^/L	9 (40.91%)	32 (46.38%)	0.654
HGB < 120 g/L	17 (77.27%)	39 (56.52%)	0.081
PLT < 100 × 10^9^/L	5 (22.73%)	6 (8.70%)	0.167
ALT > 40 U/L	8 (36.36%)	31 (44.93%)	0.480
AST > 40 U/L	8 (36.36%)	20 (28.99%)	0.514
ALP > 120 μmol/L	17 (77.27%)	45 (65.22%)	0.291
GGT > 60 U/L	18 (81.82%)	53 (76.81%)	0.843
TBIL > 17 μmol/L	11 (50.00%)	34 (49.28%)	0.953
ALB < 35 g/L	18 (81.82%)	49 (71.01%)	0.317
PT > 17 s	2 (9.09%)	8 (11.59%)	0.949
APTT > 45 s	3 (13.64%)	12 (17.39%)	0.934
BUN > 7.2 mmol/L	3 (13.64%)	12 (17.39%)	0.934
Cr > 97 μmol/L	0 (0.00%)	6 (8.70%)	0.348
Abscess diameter (cm)	6.20 (1.00–16.10)	5.45 (0.60–13.10)	0.271
Abscess number			
Solitary abscess	21 (95.45%)	46 (66.67%)	**0.008**
Multiple abscess	1 (4.55%)	23 (33.33%)	
Gas forming	5 (22.73%)	12 (17.39%)	0.806
Abscess site			
Both left and right	4 (18.18%)	7 (10.14%)	0.353
Left lobe	3 (13.64%)	7 (10.14%)	
Right lobe	14 (63.64%)	43 (62.32%)	
Other sites	1 (4.55%)	12 (17.39%)	
Pus culture results			
*Klebsiella pneumonia*	2 (9.09%)	19 (27.54%)	0.074
*Escherichia coli*	2 (9.09%)	6 (8.70%)	0.707
*Enterococcus spp.*	0 (0.00%)	2 (2.90%)	1.000
Multiple bacteria	3 (13.64%)	4 (5.80%)	0.458
Treatment			
Percutaneous drainage	9 (40.91%)	35 (50.72%)	0.680
Surgical drainage	1 (4.55%)	5 (7.25%)	
Conservative treatment	12 (54.55%)	29 (42.03%)	
Antibiotic use			
The 3^rd^ generation of cephalosporin	11 (50.00%)	40 (57.97%)	0.512
Carbapenems	6 (27.27%)	36 (52.17%)	**0.041**
Nitroimidazoles	5 (22.73%)	19 (27.54%)	0.656
Fluoroquinolone	1 (4.55%)	7 (10.14%)	0.707
Glycopeptide	3 (13.64%)	2 (2.90%)	0.165
Outcomes			
Cured	14 (63.64%)	51 (73.91%)	0.466
Improved	4 (18.18%)	11 (15.94%)	
Useless	4 (18.18%)	7 (10.14%)	
Time for temperature normalization (days)	5 (0–17)	4 (0–28)	0.922
Hospital stay (days)	12 (2–40)	12 (2–52)	0.782
Total Hospitalization expenses (dollars)	2230.74 (341.72–29,275.83)	3491.47 (665.72–15,314.91)	0.634
Retreatments in 3 months	2 (9.90%)	0 (0.00%)	0.056

### Effect of conservative treatment on PLA patients with BS history

The data presented in Table 4 demonstrates a significantly higher proportion of patients in the BS group who underwent conservative treatment compared to the other group. Therefore, we conducted a comparative analysis between conservative and non-conservative treatments in patients with a history of BS, aiming to conduct an in-depth investigation into the impact of conservative treatment on this patient population. In Table 6, no significant association was observed between receiving conservative treatment and the indicators of liver and kidney function, coagulation, complications, time for temperature normalization, hospital stay, total hospitalization expenses, retreatment in 3 months, and patient cure or improvement under similar age, gender and underlying conditions. However, within the BS group, patients undergoing conservative treatment were found to have a lower likelihood of presenting abnormally low white blood cell levels (*P* = 0.021) and low hemoglobin levels (*P* = 0.007).

**Table 6:** Comparison of conservative and non-conservative treatment in PLA patients with BS history

Variables	Non-Conservative treatment (*n* = 51)	Conservative treatment (*n* = 40)	*P* value
Age (≥ 60)	31 (60.78%)	16 (40.00%)	0.049
Gender (male/female)	22 (43.14%)/29 (56.86%)	20 (50.00%)/20 (50.00%)	0.515
Underlying conditions			
Diabetes mellitus	25 (49.02%)	20 (50.00%)	0.926
Cardiovascular diseases	3 (5.88%)	2 (5.00%)	0.779
Laboratory tests after therapy			
WBC > 10 × 10^9^/L	29 (56.86%)	13 (32.50%)	**0.021**
HGB < 120 g/L	36 (70.59%)	21 (52.50%)	**0.007**
PLT < 100 × 10^9^/L	7 (13.73%)	5 (12.50%)	0.864
ALT > 40 U/L	25 (49.02%)	15 (37.50%)	0.272
AST > 40 U/L	20 (39.22%)	9 (22.50%)	0.089
ALP > 120 μmol/L	34 (66.67%)	27 (67.50%)	0.933
GGT > 60 U/L	41 (80.39%)	30 (75.00%)	0.538
TBIL > 17 μmol/L	28 (54.90%)	17 (42.50%)	0.240
ALB < 35 g/L	40 (78.43%)	27 (67.50%)	0.240
PT > 17 s	5 (9.80%)	5 (12.50%)	0.944
APTT > 45 s	10 (19.61%)	5 (12.50%)	0.364
BUN > 7.2 mmol/L	11 (21.57%)	5 (12.50%)	0.259
Cr > 97 μmol/L	4 (7.84%)	2 (5.00%)	0.907
Complications	26 (50.98%)	14 (35.00%)	0.127
Spontaneous rupture	1 (1.96%)	0 (0.00%)	1.000
Portal vein thrombosis	0 (0.00%)	2 (5.00%)	0.190
Pulmonary infection	2 (3.92%)	1 (2.50%)	0.830
Pleural effusion	16 (31.37%)	8 (20.00%)	0.222
Abdominal effusion	7 (13.73%)	3 (7.50%)	0.545
Time for temperature normalization (days)	6 (0–19)	2 (0–28)	0.224
Hospital stay (days)	14 (3–33)	11 (2–52)	0.403
Total hospitalization expenses (dollars)	3491.47 (410.49–29,275.83)	3145.98 (341.72–18,535.12)	0.129
Retreatment in 3 months	1 (1.96%)	1 (2.50%)	1.000
Cured and improved	46 (90.20%)	34 (85.00%)	0.450

## Discussions

It is widely acknowledged that the occurrence of pyogenic liver abscess is closely associated with microbial contamination of the liver. Consistent with previous research findings, our hypothesis posits that the mechanisms underlying PLA formation following biliary surgery can be broadly categorized as follows. The first factor pertains to alterations in hepatobiliary anatomy subsequent to BS, wherein cases involving abnormal bilioenteric communication and bacterial reflux from the digestive tract often exhibit a correlation between the abscess cavity and the biliary tree [3]. The aforementioned anatomical alterations have been demonstrated to result in the development of bacterial liver abscesses through the translocation of bacteria between the hepatic parenchyma and intestinal tract [10]. Additionally, cholestasis and elevated intrabiliary pressure subsequent to BS can result in the reflux of bile containing microorganisms into the hepatic vessels [6]. Biliary strictures, including hepaticojejunostomy, serve as primary risk factors for biliary tract infection and PLA [7]. Some human errors during biliary processes like retained bile duct stone and blocked T-tube also cause cholestasis and help bacterial breeding in the bile. The third aspect can be broadly categorized as injuries occurring during biliary operations. For example, BS may irritate or even damage the mucosa of the bile ducts, compromising their defense against pathogens and leading to cholangitis followed by subsequent PLA. As suggested by Tachopoulou et al., hepatic artery thrombosis further increases the incidence of PLA through liver ischemia-related infection [11]. The broken balance between immunity and pathogens after BS is also a contributing factor. Patients undergoing BS may have compromised immune systems during the recovery period, and some may also have pre-existing diabetes mellitus, further weakening their body’s defenses. Additionally, when patients become infected and undergo emergency biliary operations, bacteria from the primary infection site can enter the bloodstream and lead to PLA. While bowel preparation alone cannot prevent PLA following biliary endoscopic procedures, effective bowel cleansing may help reduce the incidence of PLA in patients with biliary endoscopic interventions [12].

Types of BS also significantly influences the development of PLA. The occurrence of PLA is more commonly observed in BEA and indwelling biliary endoprosthesis compared to other BS [2]. In fact, BEA itself can even be treated as a major independent risk factor [9, 13, 14]. We believe that this is due to a mechanism mentioned in the first category whereby abnormal bilioenteric communication resulting from BEA, hepaticojejunostomy procedures, and indwelling biliary stents leads to the reflux of bowel bacteria into the intrahepatic bile ducts [2, 3, 15].

In this study, no significant correlation was observed between aging and a history of BS in the PLA. However, prior to excluding patients with tumors, it was noted that a higher proportion of patients with a BS history were aged ≥ 60 years, which aligns with the notion that the incidence of malignant diseases increases with age [16]. Patients in the BS group exhibited a higher incidence of pleural effusion, which we hypothesized to be attributed to infection and subsequent emergency biliary operation. PLA patients with a history of BS demonstrated a greater likelihood of being female compared to those without BS, potentially due to elevated estrogen levels leading to cholestasis, resulting in increased TBIL levels and the development of cholecystolithiasis or calculus of bile duct. Both the BS group and the BEA group were more prone to developing diabetes mellitus, serving as another widely acknowledged risk factor for cholecystolithiasis and calculus of bile duct. The possible explanation for these phenomena is that diabetes mellitus, which is associated with an immunocompromised state, increases the incidence of bacterial contamination in the liver after BS, particularly due to abnormal bilioenteric communication caused by BEA [4]. Additionally, we observed an increase in abnormalities of TBIL and coagulation in the BS group, which are considered complications of this type of surgical procedure [17].

It is noteworthy that the BS group exhibited higher revenue in *pus* culture, while the negative ratio was comparable between the two groups in blood culture [18]. The presence of pathogens in the liver following biliary surgery suggests a weakened bile duct defense mechanism, allowing for their migration from the intestines. These pathogens then disseminate and propagate along the bile duct and capillary bile duct wall through reflux. Interestingly, our findings indicate that the BS group experience similar gas-forming conditions compared to patients without BS history but exhibit lower levels of *Klebsiella pneumonia* in blood culture results. This contradicts previous beliefs suggesting that *Klebsiella pneumonia* could cause gas-forming conditions in PLA [19]. The possible explanation may be the shift in flora from *Klebsiella pneumonia* to other gas-producing bacteria in the hepatobiliary system following biliary surgery. Although *Klebsiella pneumonia* infection is reported as the primary pathogen of multiple PLA, we observed no significant difference in its incidence between the BEA and non-BEA groups; however, we did find more solitary abscesses in the former group compared to the latter [20].

We observed that the BS group exhibited elevated levels of WBC and HGB abnormalities, which aligns with the prevailing notion that non-conservative therapy is efficacious in managing BS. Surgical drainage is deemed appropriate for abscesses originating from the biliary system, cases involving bile duct obstruction, ruptured lesions, as well as large abscess cavities characterized by thick walls or septa [21]. The selection between percutaneous or surgical drainage becomes more intricate due to the specific structural changes in patients’ hepatobiliary system following surgery. For example, Sugiyama et al. maintained that percutaneous drainage was the optimal approach for treating PLA, with additional interventions to alleviate obstruction, while cases involving biliary communication necessitated endoscopic biliary stent placement [22]. The authors also acknowledged the limitations of percutaneous drainage in patients with biliary-related problems, thus advocating for surgical intervention as a more effective approach [15, 23]. In clinical treatments for uncomplicated PLA without BS history, conservative management options such as systemic supportive care and administration of antibiotics or diuretics alone are preferred for multiple or small abscesses with a diameter below 5 cm. However, larger single abscesses with an evident hypoechoic area in the liver may benefit from percutaneous drainage intervention [24]. The majority of abscesses in our cases exceeded the size mentioned above, thus percutaneous drainage remained the predominant therapeutic approach in the non-BS group.

Surprisingly, we observed that the BS group received more conservative treatment with reduced utilization of surgical drainage. This can be attributed to their compromised health status, including impaired immunity, elevated TBIL levels, and coagulation disorders. These factors may account for the lower rate of improvement and higher incidence of inconclusive reports among patients in the BS group, as previously suggested by other researchers [18]. Experimental antibiotics targeting gram-negative bacilli of biliary origin, including 3^rd^ generation cephalosporins, fluoroquinolones, and carbapenems, exhibit differential usage patterns between patients with BS and those without BS history [25]. Specifically, the utilization of 3^rd^ generation cephalosporins and fluoroquinolones is more prevalent in the BS group due to their high concentration in bile. In the subgroup of patients with BEA, carbapenem appeared to be more commonly prescribed compared to other groups, possibly due to compromised immunity in the BEA group. This may lead to resistance against 3^rd^ generation cephalosporins and fluoroquinolones but sensitivity towards carbapenems for certain gram-negative bacillus infections. A previous study also highlighted that PLA patients with BEA history who experienced ablation-related fever primarily developed *Enterobacter* and *E. faecalis/E. faecium*. infections, which are susceptible to carbapenems [12].

In this study, the most apparent limitations can be briefly outlined as follows. Firstly, it is important to acknowledge that being a single-center study renders it less robust in terms of external validity compared to multi-center studies. Nevertheless, the results still possess significant generalizability and can serve as a valuable reference for other researchers and clinicians due to the substantial sample size and our extensive expertise in diagnosing and treating PLA patients with BS history in northwestern China. Moreover, an extended follow-up period is required, considering that we did not collect data on mortality rate or conduct multivariate analysis to examine risk factors for short-term survival since our recent study had a limited follow-up duration of only 3 months.

## Conclusions

The clinical characteristics and treatments exhibit significant variations between PLA patients with and without BS history. The BS group demonstrate elevated levels of TBIL along with impaired coagulation function. Additionally, they are more susceptible to *Escherichia coli* infection and multiple bacterial infections, but less likely to be affected by *Klebsiella pneumonia*. The efficacy of treatments was found to be comparatively lower in the BS group. However, these patients tended to receive more conservative treatment, particularly those with mild symptoms and clinical features. Patients who received the BEA group exhibited a higher prevalence of diabetes mellitus, as well as elevated WBC but decreased HGB levels. Consequently, having a history of BS, especially when treated with BEA, contributes to the development of PLA in patients with poor baseline conditions, but exerting no influence on their prognosis.

## Data Availability

The datasets used and/or analysed during the current study available from the corresponding author on reasonable request.

## Abbreviations

PLA: Pyogenic liver abscess
WBC: White blood cell
ALT: Alanine aminotransferase
AST: Aspartate aminotransferase
TBIL: Total bilirubin
ALB: Albumin
PT: Prothrombin time
APTT: Activated partial thromboplatin time
BUN: Blood urea nitrogen
Cr: Creatinine
DM: Diabetes mellitus
Non-DM: Non-diabetes mellitus
